# Publication rates of research presented at the Canadian Society of Otolaryngology-Head and Neck Surgery Annual Meetings from 2008 to 2018: an 11-year review

**DOI:** 10.1186/s40463-022-00606-5

**Published:** 2023-02-08

**Authors:** Aileen M. Feschuk, Kalpesh Hathi, S. Mark Taylor

**Affiliations:** 1 Faculty of Medicine, Memorial University of Newfoundland, 300 Prince Philip Dr., St. John’s, NL A1B 3V6 Canada; 2 Faculty of Medicine, Dalhousie Medicine New Brunswick, Saint John, NB Canada; 3 Division of Otolaryngology - Head and Neck Surgery, Department of Surgery, Dalhousie University, Halifax, NS Canada

**Keywords:** Publication rate, Knowledge translation, Otolaryngology, Conference, CSOHNS

## Abstract

**Background:**

Knowledge dissemination is paramount so physicians may practice the most up-to-date, evidence-based medicine to best serve their patients. Medical conferences are a commonly employed method of facilitating this. By determining the publication rate of research presented at a conference, the quality of the conference is indirectly assessed. Therefore, this study aimed to determine the publication rate, along with other conference metrics, of abstracts presented at the Canadian Society of Otolaryngology-Head and Neck Surgery (CSOHNS) meetings from 2008 to 2018.

**Methods:**

All abstracts presented at the CSOHNS Annual Meetings from 2008 to 2018 were reviewed from publicly available records. Presentation year, presentation type (i.e. oral or poster), whether each abstract was presented in the Poliquin Resident Research Competition, and the country in which the lead author’s institution was located, were collected. Publication status of each abstract was then determined using a six-tiered search strategy in PubMed and Google Scholar. All data were then analyzed using SPSS Version 27.0.

**Results:**

From 2008 to 2018, 1947 abstracts were analyzed, yielding an overall publication rate of 58.7%. There was a significantly increasing trend in publication rate over the 11 years (p = 0.015). The rate of publication differed based on type of presentation (oral 65.1%, poster 50.2%; p = 0.001). Most presentations were presented by a first author associated with a Canadian institution (94.4%). The top journal in which research was published was Journal of Otolaryngology- Head and Neck Surgery (37.3%). The mean impact factor of the journals in which presentations were published was 2.92. Finally, the median time to publication was 14 months (IQR: 9.0–25.0).

**Conclusions:**

Research presented at 2008–2018 CSOHNS annual meetings was published in academic journals at higher rates than research at comparable conferences. Oral presentations have a significantly greater publication rate, compared to poster presentations. Additionally, the upward trend in publication rate over the 11 meetings suggests a steady increase in the quality of research being presented.

**Graphical abstract:**

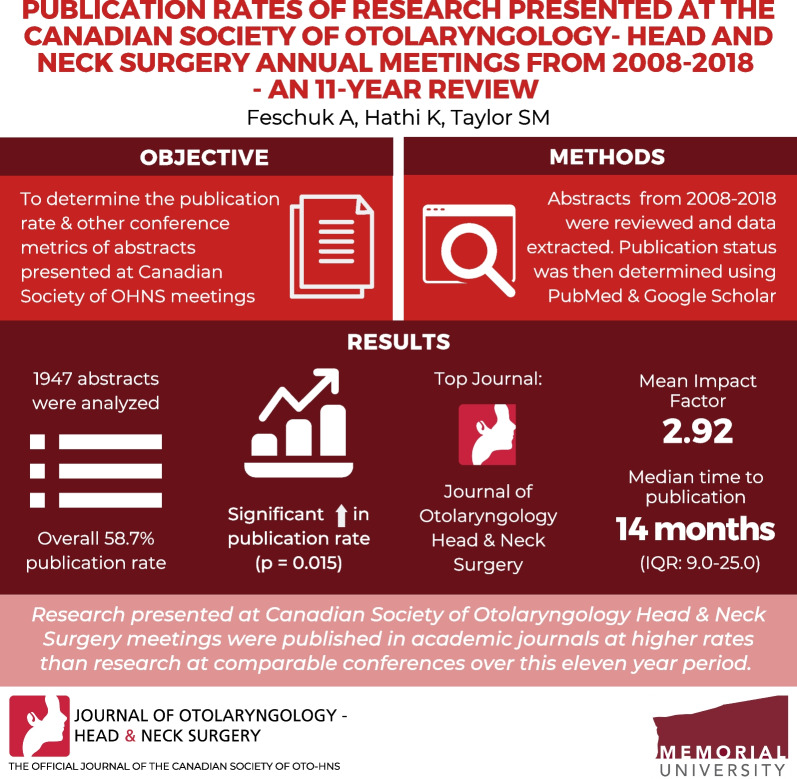

## Background

One of the primary purposes of medical conferences is to facilitate communication between practicing physicians, residents, and medical students so that they may explore issues within a specialty, analyze results, and offer potential solutions with the goal of maximizing patient care [1]. The CanMEDS framework, designed and promoted by The Royal College of Physicians and Surgeons of Canada (RCPSC), identifies abilities physicians must have to properly address the healthcare needs of their patients. Some of these competencies suggest physicians should share and compare their work, while seeking and providing feedback through collaborative learning and knowledge dissemination to improve personal and collective practice [2]. These competencies outlined by the RCPSC highlight the importance of having high quality, specialty specific conferences available for physicians to attend and present their findings.

By determining the publication rate of research presented at a conference, the quality of the conference is indirectly assessed [3]. Conferences in various countries and across multiple specialties have previously assessed the publication rates of research presented at their meetings [3–10]. Publication rates at various Otolaryngology—Head and Neck Surgery conferences internationally range from 19 to 56.8% [4–9]. A 2018 Cochrane review of research assessing the publication rate of biomedical conferences, exploring 425 reports, and over 300 000 abstracts, found an overall publication rate of 37.3% [10]. These previous studies suggest that a large proportion of research presented at scientific conferences, including those in the field of Otolaryngology—Head and Neck Surgery, do not undergo or pass the peer review process for dissemination in the scientific literature. Therefore, knowledge of publication rates of research presented at academic conferences may provide an indirect measure of the quality of the work presented at these meetings.

In September 2021, the Canadian Society of Otolaryngology-Head and Neck Surgery (CSOHNS) hosted its 75th Annual Meeting. However, the only assessment of this conference’s publication rate was completed in 2014 by Ogilvie et al. [11] who investigated the publication rate of oral presentations at the CSOHNS annual meetings from 2006 to 2010. These authors found that 50.5% of oral presentations were published [11]. However, this study excluded poster presentations from the analysis and therefore could not compare the publication rates of poster versus oral presentations. Therefore, we aimed to expand on this work and update the publication rate data for the annual CSOHNS meeting over the past decade.

This manuscript identifies the overall publication rate of all research presented at the annual meeting of the CSOHNS from 2008 to 2018. This manuscript also compares the publication rates of oral and poster presentations over the 11-year study period. The Poliquin Resident Research Competition was also described as a subgroup of the oral presentations. The time from presentation to publication along with the top journals published in and the corresponding impact factors of those journals is also reported.

## Methods

### Data collection

All abstracts presented at the CSOHNS Annual Meetings from 2008 to 2018 were collected from the CSOHNS publicly available website records [12]. Data including year of presentation, whether each abstract was presented orally or via poster, whether each abstract was presented in the Poliquin Resident Research Competition, and the country in which the lead author’s institution was located, were collected.

**Fig. 1:**
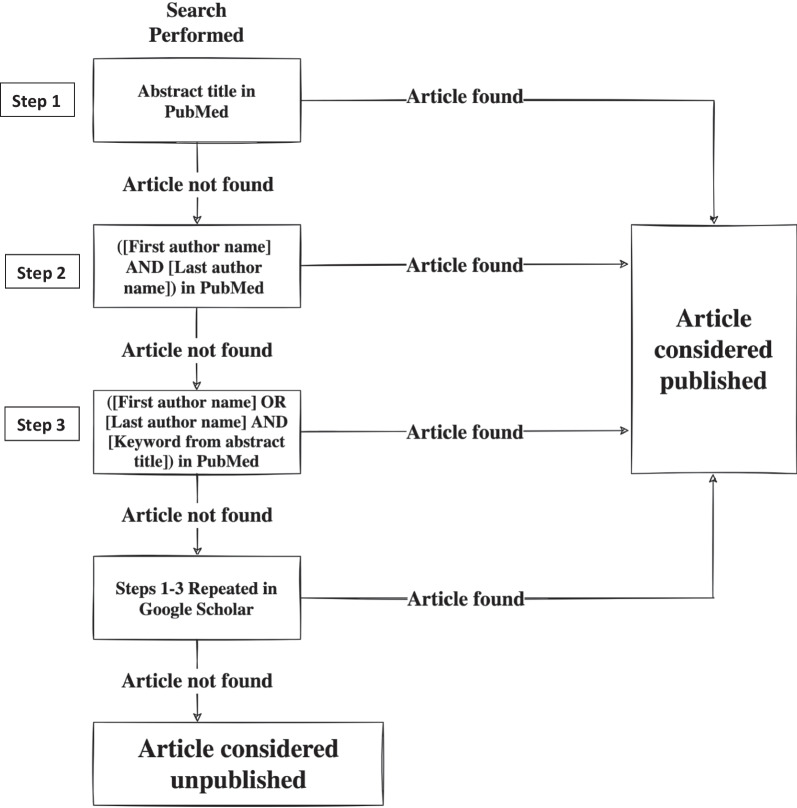
Search strategy used to determine publication status of abstract presentations at CSOHNS Annual Meeting (2008–2018)

The publication status of the presentations was then determined using the following search strategy: The abstract title was searched in PubMed. If the article was not found on the first page of hits, a search using the first and last authors’ names were searched in PubMed. If again the article was not found on the first page of hits, a search using the first or last authors’ names and a keyword from the abstract title were searched in PubMed. Keywords were considered words which, standalone, gave context regarding the article’s main focus (e.g. for the title *Current Management of Epistaxis,* a keyword would be considered epistaxis). If the article was not found on the first page of hits, the previous steps were repeated in Google Scholar. If no article was found using this protocol, the abstract was considered unpublished. This protocol can be visualized in Fig. 1. An abstract was considered published if an article discussing the same topic as the abstract was found in a peer reviewed journal and was written by at least two of the same authors as the abstract. The exception was for abstracts with only one or two authors, in these situations only one authors’ name was required on the peer-reviewed publication. Once an abstract was considered published, the journal, journal impact factor according to the Clarivate database, and the date of publication were recorded [13]. If the journal impact factor was not found within the Clarivate database, it was considered not to have one and was excluded in the impact factor descriptions. Additionally, the impact factors used were the most recently updated values in Clarivate, not the impact factor at the time of publication.

Data extractions were completed up to and including the 2018 meeting. Data was extracted in January 2022 allowing 33 months for the research from the latest June 2018 meeting to reach publication. The previous analysis of the publication rate of oral presentations at the CSOHNS meetings from 2006 to 2010 found that the average time to publication was 21 months [11]. This suggests 33 months should be sufficient to capture most of the research that would be published.

### Data analysis

Only papers published within the same month as, or after, their respective meeting were included in the analysis for time from publication to presentation. All papers published before presentation were excluded as this would have biased results by including negative time from presentation to publication values. These papers were included in all other analyses. Only papers published in journals with impact factors available in the Clarivate database were included in the impact factor analyses.

The number of presentations, publication rates, time from presentation to publication (in months), and impact factor of the journal published in was reported descriptively. Each variable was described for all articles presented, and as well independently by presentation type (i.e. poster versus oral). The Poliquin Residents Research Competition presentations were considered oral presentations but were also described independently regarding each variable.

The time from presentation to publication was described as a median value, along with interquartile ranges (IQR). The frequency at which presentations were published in individual journals was described as a percentage and the country of the lead author’s home institution was also described as percentages. The impact factors were described as means ± standard deviation (SD). Each step in the search strategy from Fig. 1 was labeled 1–3, the percentage of publications found at each search tier was reported.

The publication rate of oral versus poster presentations at the CSOHNS conferences over our 11-year review was compared using a 2 × 2 chi-square test. The time from presentation to publication in months, for oral versus poster presentations over the 11-year review period was compared using a Mann–Whitney U test as the data was not normally distributed (Shapiro Wilk, p < 0.001). Cochran-Armitage tests for trend (chi-square test for trend) were used to identify increasing publication trends over the study period (2008–2018) for all presentations, and individually for oral and poster presentations.

All data was analyzed using SPSS Version 27.0 and R version 4.1.0 (package DescTools 0.99.42). A priori power analysis using GPower (version 3.1.9.7) was performed for all statistical analyses (conservative small effect size, chi-square test: effect size: 0.1; alpha: 0.05, power 0.8, df: 1, n = 785 & t-test (for Mann–Whitney analyses): effect size: 0.2, alpha: 0.05, power: 0.8, allocation ratio: N2/N1: 1, two-tailed test, n = 788) and indicated that the 1947 abstracts included in this study would sufficiently power the analyses. A priori significance level of p < 0.05 was set for all analyses.

## Results

**Fig. 2:**
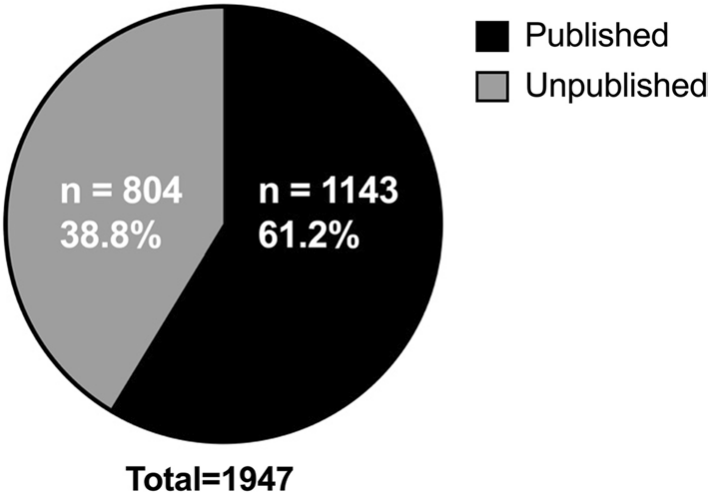
Pie graph representing published and unpublished research presented at the 2008–2018 CSOHNS annual meetings

**Fig. 3:**
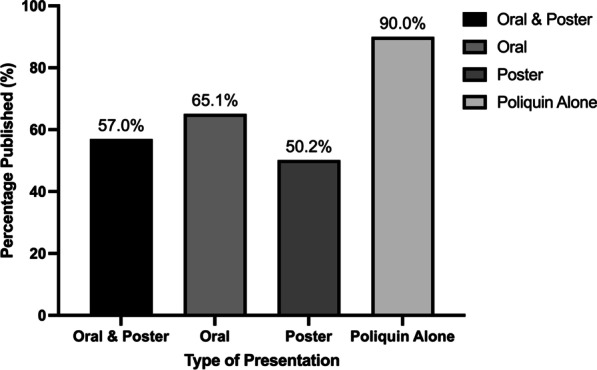
Publication rates of presentations at the 2008–2018 CSOHNS annual meetings, based on presentation type

A total of 1947 abstracts were presented at the 2008–2018 CSOHNS annual meetings. Overall, the percentages of publications identified at each tier of the search strategy from 1 to 6 were 64.7% (n = 739), 3.0% (n = 34), 4.0% (n = 46), 18.8% (n = 215), 3.2% (n = 37), and 6.3% (n = 72) respectively. Of these identified abstract presentations, 837 (43%) were poster presentations, and 1110 (57%) were oral presentations. In total, 1143 presentations were identified as published through the outlined search strategy (Fig. 1), for an overall publication rate of 58.7% (Figs. 2 and 3). Most abstracts were presented by a first author associated with a Canadian institution (94.4%). The top five countries represented at the conferences over the 11-year period were (1) Canada (94.4%, n = 1837); (2) Saudi Arabia (2.0%, n = 39); (3) United States (1.7%, n = 34); (4) United Kingdom (0.7%, n = 14); (5) India (0.4%, n = 8).

The rate of publication of the oral presentations was 65.1%, whereas the publication rate of the poster presentations was 50.2% (Fig. 3). The 2 × 2 chi square test comparing the publication rate of the oral versus poster presentations found that a greater proportion of oral presentations were published compared to poster presentations (65.1% vs. 50.2%; Χ^2^(1) = 44.03, p < 0.001). Over the 11 meetings, a total of 170 or 8.7% of presentations were part of the Poliquin Resident Research Competition, of which, 153 (90%) were published (Fig. 3).

Overall, the top five journals in which research from the 11 conferences were published, in descending order, were: (1) Journal of Otolaryngology- Head and Neck Surgery (37.3%, n = 425); (2) Laryngoscope (6.3%, n = 72); (3) Otolaryngology- Head and Neck Surgery (5.4%, n = 61); (4) Head and Neck (4.4%, n = 50); and (5) International Journal of Pediatric Otorhinolaryngology (4.3%, n = 49). The top five journals in which specifically oral presentations were published followed the same order as overall, with rates of 41.8% (n = 301), 7.8% (n = 56), 6.1% (n = 44), 4.6% (n = 33%), and 3.6% (n = 26), respectively. The top five journals in which poster presentations were published, in descending order, were: (1) Journal of Otolaryngology- Head and Neck Surgery (29.5%, n = 124); (2) International Journal of Pediatric Otorhinolaryngology (5.5%, n = 23); (3) and (4) Head and Neck and Otolaryngology-Head and Neck Surgery (both 4.0%, n = 17); and (5) Laryngoscope (3.8%, n = 16). The oral and poster presentations were published in a total of 186 different journals. According to the Clarivate database, the mean impact factor of the journals in which all presentations, oral presentations, and poster presentations were published was 2.92, 3.00, and 2.72, respectively. This only included journals in which the impact factor was available. Overall, 77/186 (41.4%) of the journals had impact factors available on the Clarivate database, therefore 143/1143 (12.5%) of papers were published in journals without impact factors.

**Fig. 4:**
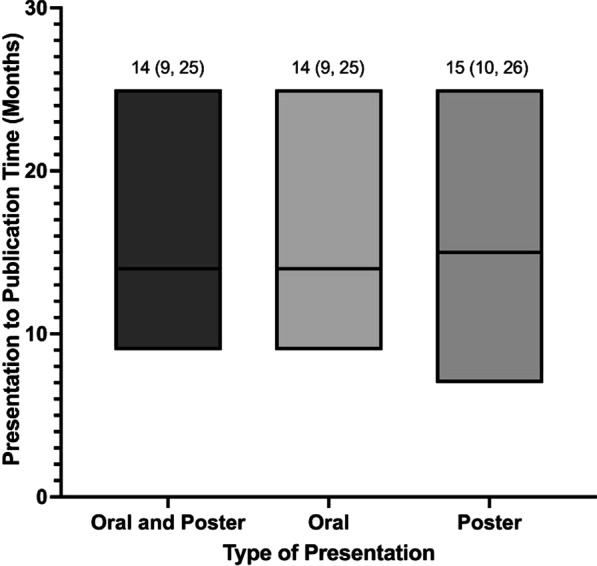
Median and interquartile ranges of time from presentation to publication from 2008 to 2018 CSOHNS meetings

The median time from presentation to publication of all (oral and poster) presentations, and oral presentations only, which were published after presentation was 14 months (IQR: 9.0–25.0). The median time from presentation to publication for only poster presentations was 15 months (IQR: 7.0–25.0) (Fig. 4). The Mann–Whitney U showed no significant difference in time from presentation to publication between oral and poster presentations (U = 109,920.0, z = − 0.388, p = 0.698).

**Fig. 5:**
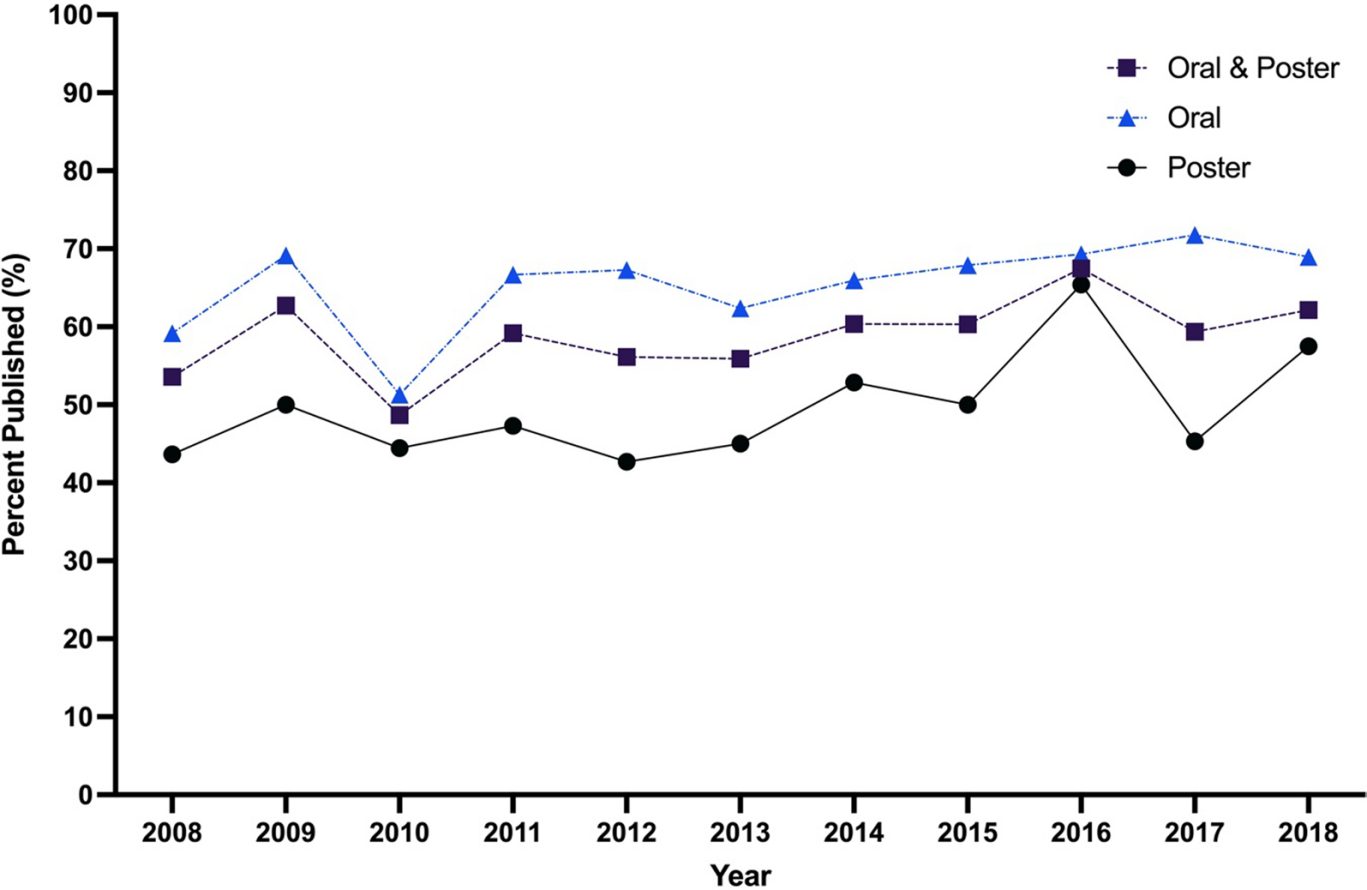
Year-to-year publication rates based on presentation type at the 2008–2018 CSOHNS annual meetings

The Cochran-Armitage test for trend assessing the publication rates from 2008 to 2018 showed a significant increase in publication rates for all presentations (Χ^2^(1) = 5.98, z = − 2.45, p = 0.015), poster presentations (Χ^2^(1) = 5.92, z = − 2.43, p = 0.015) and oral presentations (Χ^2^(1) = 5.19, z = − 2.28, p = 0.023) (Fig. 5).

## Discussion

Conferences among medical professionals are a widely accepted setting to facilitate dissemination of important research findings, and it is therefore important that high quality conferences, where practitioners and learners may attend and present their research, exist. The publication rate of research presented at conferences indirectly reflects the quality of the conference [3]. This study identified the overall publication rate of all abstracts presented at the annual meeting of the CSOHNS from 2008 to 2018. We also determined and compared the publication rates and mean time from presentation to publication, along with the most common journals authors published in and the mean impact of these journals for oral, poster, and Poliquin Resident Research Competition abstracts over the 11-year study period.

This analysis found that abstracts presented at the 11 annual meetings had an overall publication rate of 58.7% (n = 1143). The publication rates of five international Otolaryngology conferences reported in the literature ranged from 19 to 56.3% (Table 1) [4–9]. Further, a 2018 Cochrane systematic review found a publication rate at biomedical conferences to be 37.3% [10]. As the publication rate of the CSOHNS meeting exceeds these values, it indirectly suggests that research presented at the CSOHNS annual meetings is of high quality, with a publication rate higher than comparable conferences. However, 41.3% (n = 804) of presentations were not published, and therefore, conference attendees should continue to apply a critical lens to their personal assessment of research presented at medical conferences. Further, most presentations would not have undergone the peer-review process prior to presentation, unless the research was published before the respective annual meeting. That is to say, while abstracts submitted to conferences are reviewed by medical committees, they are not usually considered peer-reviewed to the same standards that publications are. Of course, published or not, one should always apply a critical lens when evaluating research.

**Table 1:** Publication rates of Otolaryngology conferences internationally

Conference	Years Assessed	Publication Rate (%)
*Canadian Society of Otolaryngology-Head and Neck Surgery (Current study)*	*2008–2018*	*58.7*
Annual Meeting of the American Academy of Otolaryngology – Head and Neck Surgery [4]	1993–1995	32
Otorhinolaryngological Research Society Meetings [6]	1996–2015	56.3
British Academic Conference in Otolaryngology [7]	2009	24
British Academic Conference in Otolaryngology [7]	2012	19
British Association of Head and Neck Oncologists’ Meetings [9]	2009–2015 (excluding 2014)	31.1
ENT Scotland’s Annual Meetings [8]	2005–2014	50.3

Our data also demonstrated a significant difference in the publication rate based on presentation type, with oral presentations having a publication rate of 65.1% (n = 723) and poster presentations having a publication rate of 50.2% (n = 420). This is also consistent with what was found by the 2018 Cochrane review investigating conference publication rates [10]. They found that, from 143 reports and over 115 000 abstracts, oral presentations were associated with full publication more than poster presentations [10]. In fact, the relative risk of publication of oral presentations compared to poster presentations was 1.46. This is expected, as typically oral presentations are reserved for the research considered to be of higher quality, impact, and greater interest for the conference attendees, and is therefore more challenging to secure compared to poster presentations. In fact, when submitting abstracts to many conferences, including this one, authors will often have to select the option to be considered for a poster presentation if their work is not accepted for oral presentation. Further, previous literature has shown that papers with positive research findings are overall more likely to be accepted for presentation at biomedical meetings [10, 14].

Our data demonstrated a significantly increasing trend in publication rate over the 11-year period. This suggests an increase in the quality of research presented at annual meetings over the 11-year period. However, the increase in publication rate is likely multifactorial but could be in part due to the increased demand for medical students and residents to engage in research. Most medical schools and residency programs in Canada have research requirements integrated into their curriculum [15]. Aside from curriculum requirements, medical students may be engaging in more research because the Canadian Resident Matching Service (CaRMS) has become more competitive recently, with only 28 Canadian medical graduates going unmatched in 2008, compared to 172 Canadian medical school graduates going unmatched in 2018 [16]. In addition, it has been found that among a group of 312 Canadian Otolaryngologists, the mean publication rates both before and during residency increased significantly from 1998 to 2013 [15]. In fact, the CSOHNS annual meetings’ Poliquin Resident Research Competition, is an annual research competition that is highly regarded across the country and is specific to resident research. Interestingly, Cochrane reviews have not found an increasing overall publication rate of abstracts presented at biomedical conferences despite the increase in online medical journals [10, 17]. This suggests that this may be a finding unique to this conference, rather than an overall increase in publication rates across biomedical literature.

The CSOHNS states that some goals of the Poliquin Resident Research Competition are to “foster high quality research and innovation as an important element of residency training”, and “to highlight impactful research by trainees” [18]. It is of relevance then, that abstracts presented in this competition had a 90% publication rate. However, it should be noted that submission for consideration into the Poliquin Resident Research Competition requires assessment of a full and complete manuscript. This is significant, as a previous analysis of oral presentations from the CSOHNS annual meetings from 2006 to 2010, found that the most common reasons given by authors for not having published research presented at the conference was that the “research is still in progress” and that the trainee working on the project had “moved on” [11]. It is important to keep this in mind when interpreting the rate of oral presentation publication, as research from this residents’ competition was treated as a subset of this.

It is also noteworthy that the first author of 94.4% (n = 1837) of abstracts overall (96.4% (n = 1070) oral, 91.6% (n = 767) poster) was representing a Canadian institution. Further, the Journal of Otolaryngology- Head and Neck Surgery (the official journal of the CSOHNS) was the journal in which the highest frequency in which abstracts were published. It is, however, important to note, some meetings’ Poliquin Resident Research Competitions requested residents submit their manuscript to the Journal of Otolaryngology- Head and Neck Surgery as part of the competition. According to the Clarivate database, the average impact factor of journals in which abstracts were published was 2.92. However, it is important to note that 12.5% (n = 143) of published studies were in journals without an impact factor reported in Clarivate, and these were therefore excluded from the calculation of the descriptive statistics surrounding impact factor. Nevertheless, this demonstrates a large quantity of high-quality research conducted at Canadian institutions, are presented at this Canadian conference, and published in this Canadian journal.

Finally, the median time to publication, both overall and for oral presentations only, was found to be 14 months (IQR: 9.0–25.0) and was 15 months (IQR: 7.0–25.0) for poster presentations. This is similar to the average time to publication for oral presentations at the 2006–2010 CSOHNS annual meetings, which was 21 months [11]. The time between the 2018 conference and when data extractions for these analyses were performed was 33 months. Considering the 75^th^ percentile of time to publication was 25 months, most, but not all studies were published less than 33 months after presentation. Apart from a few outliers from the most recent years of our 11-year study period, we have likely given enough time to capture the overall pulse of the publication rate.

An important limitation to consider is that it is possible that not all publications were identified through the search strategy outlined in Fig. 1. In fact, 6.3% (n = 72) of publications were not identified until the sixth/final tier of the search strategy. However, our search strategy is similar in rigor to analyses of previous conferences publication rates, including the assessment of the CSOHNS conference from 2006 to 2010, and is therefore in line with the literature in this field [3, 11]. This limitation may be improved in future studies by including a third data base and/or including additional key words. Alternatively, future studies looking to investigate conference publication rates may consider reaching out to the corresponding authors of the conference presentations to inquire about the publication status of their work. Additionally, given that until recently, research presented in the Poliquin Resident Research competition had to be submitted to the conference as a full manuscript, this may have influenced the high rates of publication of oral abstracts. Finally, as mentioned not all journals having impact factors available on Clarivate, and the fact that the most recently available impact factor was used in this study, not the impact factor at the time of research publication, limited our analyses of the journal impact factors.

## Conclusion

In conclusion, our data suggests that the CSOHNS annual meetings consist of high-quality research, which is published at a rate higher than other conferences in the field of Otolaryngology-Head and Neck Surgery (58.7%). However, 41.3% of abstracts presented were not published, and conference attendees should continue to assess presented research with a critical lens. Additionally, oral presentations had a higher publication rate compared to poster presentations which is consistent as oral presentations tend to be more challenging to secure than poster. In general, Canadian Otolaryngologists appear to be conducting high quality research, presenting their work at the CSOHNS annual meetings, and publishing their work in this Canadian journal.

## Data Availability

The datasets used and/or analysed during the current study are available from the corresponding author on reasonable request.

## Abbreviations

CSOHNS: Canadian Society of Otolaryngology-Head and Neck Surgery
RCPSC: Royal College of Physicians and Surgeons of Canada
IQR: Interquartile range
SD: Standard deviation
CaRMS: Canadian Resident Matching Service
